# Duplex qPCR for detecting and differentiating porcine epidemic diarrhea virus GI and GII subtypes

**DOI:** 10.3389/fmicb.2025.1475273

**Published:** 2025-01-22

**Authors:** Bin Wang, Wang Han, Di Wu, Yue Jing, Li Ma, Feiyang Jiang, Shusen Ji, Lianmei Bai, Xiuju Yu, Wei Hou, Shouyu Wang, Haidong Wang

**Affiliations:** ^1^College of Veterinary Medicine, Shanxi Agricultural University, Jinzhong, China; ^2^OptiX+ Laboratory, Wuxi University, Wuxi, China

**Keywords:** quantitative real-time PCR (qPCR), porcine epidemic diarrhea virus (PEDV), PEDV GI and GII subtypes, S protein, isolated epidemic strain

## Abstract

**Introduction:**

Porcine epidemic diarrhea virus (PEDV) is a pathogen that causes a highly contagious intestinal disease in pigs, which causes significant economic losses to the pig industry worldwide. PCR is the most commonly used technique for PEDV diagnosis in practical clinics, however, reported works still suffer from shortcomings, for example, most of them cannot differentiate GI and GII subtypes, they suffer from low sensitivity, and some primer sequences are no longer able to match the mutant strains.

**Methods:**

To address these issues, we conducted a comprehensive analysis by comparing the sequences of the PEDV S protein in the existing NCBI database with a recently isolated epidemic strain of PEDV, named SX0818-2022, of subtype GIIa from Shanxi, China. The conserved sequences of GI and GII subtypes were retrieved to design the primers and probe. Leveraging this information, we developed a TaqMan probe-based quantitative real-time PCR (qPCR) assay that is uniquely tailored to detect both PEDV GI and GII subtypes.

**Results:**

Additionally, this qPCR can identify PEDV GI and GII subtypes with high sensitivities of 90 copies/μL and 40 copies/μL, respectively (refers to the number of copies of the DNA target per microliter of template in the reaction system), much higher than the previously reported works and especially suitable for early diagnosis and prevention. Besides, excellent specificity and repeatability of the duplex qPCR were verified, thus supporting its potential applications in practical clinics.

**Discussion:**

Therefore, this work presents a promising tool for PEDV diagnosis, prevention, and control.

## 1 Introduction

Porcine epidemic diarrhea (PED) is a highly contagious enteric disease in pigs, caused by the porcine epidemic diarrhea virus (PEDV) (Jung et al., 2020), which was first identified in the United Kingdom in 1971 (Chasey and Cartwright, 1978), first isolated in Belgium in 1978 (Pensaert and Bouck, 1978), and is now prevalent worldwide (Chen et al., 2014; Hanke et al., 2017; Huang et al., 2013; Jung and Saif, 2015; Kusanagi et al., 1992; Lei et al., 2024; Stevenson et al., 2013; Sun et al., 2012; Takahashi et al., 1983; Tian et al., 2014). This virus elicits symptoms including diarrhea, vomiting, decreased appetite, depression, and dehydration, affecting pigs of all ages. Piglets are particularly susceptible, with extremely high post-infection mortality rates close to 100% (Jung et al., 2015), thus resulting in huge economic losses to the pig industry. The main source of infection of PED is sick and carrier pigs, and its transmission is mainly through direct transmission, of which the fecal-oral route is the core route of direct transmission (Jung and Saif, 2015). At the same time, the indirect transmission caused by contaminated transportation tools, clothes, shoes, utensils, and feeds also plays an important role in the spread of the disease (Bowman et al., 2015; Kim et al., 2017; Lowe et al., 2014; Pasick et al., 2014).

PEDV is a single-stranded, positive-sense RNA virus that possesses an approximately 28 kb genome. This genome is characterized by the presence of non-coding regions at both its 5′ and 3′ termini, as well as 7 open reading frames (ORFs) designated as ORF1a, ORF1b, and ORF2 through ORF6. These ORFs encode a total of 17 non-structural proteins, in addition to 4 essential structural proteins: the spike (S), envelope (E), membrane (M), and nucleocapsid (N) (Lee, 2015; Qiu et al., 2022). Notably, the S protein functions as a surface immunogenic protein, involved in the binding and membrane fusion processes between the virus and its host cell receptor (Lee, 2015). Based on the evolutionary analysis of the S protein, PEDV is primarily classified into two distinct subtypes: classical (GI) and variant (GII) strains (Hanke et al., 2017). The GI subtype is further subdivided into two subtypes, GIa and GIb. The GI subtype, characterized by its relatively weaker virulence compared to the GII subtype, is predominantly found in Europe and Asia (Lin et al., 2016; Pensaert and Martelli, 2016). The GII subtype can be further subdivided into three distinct subtypes: GIIa, GIIb, and S-INDEL (Lin et al., 2022; Wang H. et al., 2020). The GIIa subtype comprises mutant strains originating from various regions, including the United States, China, and Japan (Wang D. et al., 2016). The GIIb subtype, exemplified by the AJ1102 strain, is predominantly prevalent in Asia (Wang D. et al., 2016). The S-INDEL subtype, represented by the OH851 strain, is notably less virulent and pathogenic compared to non-S-INDEL subtypes (Guo et al., 2019; Lee, 2015; Vlasova et al., 2014). The emergence of the S-INDEL subtype may be attributed to recombination events occurring between classical and mutant strains (Lee, 2015; Zhang et al., 2022). Prior to 2010, all PEDV isolates identified in China belonged to the GI subtype, but subsequently, the prevalent epidemic strain shifted to the GII subtype (Lei et al., 2024; Yu et al., 2023; Zhang et al., 2023). A comprehensive sequencing analysis of 74,568 PEDV-positive samples collected in China from 2011 to 2021 revealed 65 complete PEDV genome sequences: only one strain was classified as the GI subtype, while the remaining 64 strains belonged to the GII subtype. When these 65 strains were analyzed alongside 607 additional PEDV strains sourced from public resources, it was evident that 89.9% of the total strains were of the GII subtype, while 10.1% retained the GI subtype (Zhang et al., 2023), illustrating the significant epidemiological transition in China.

**Figure 1:**
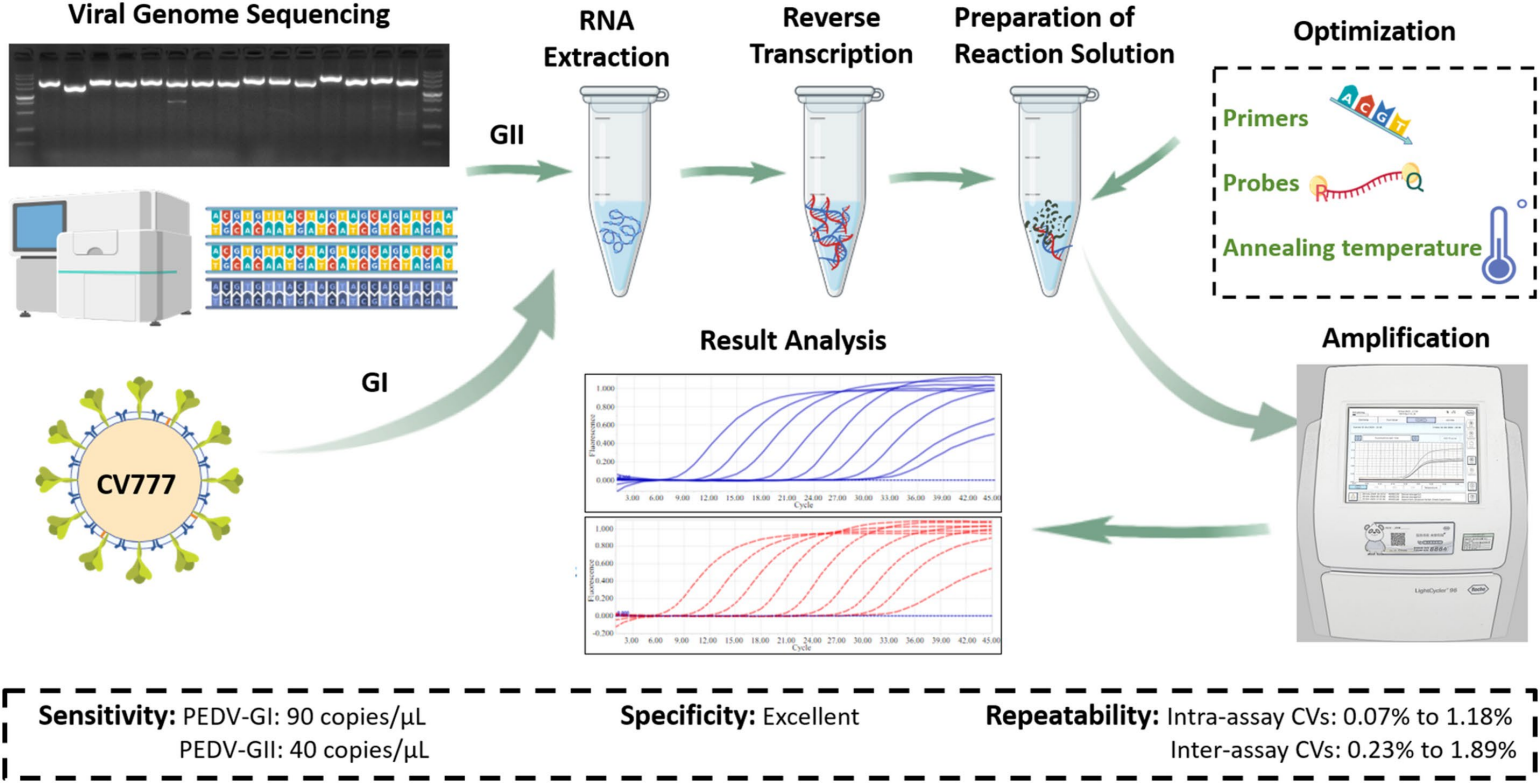
Research and development process flowchart (Created with BioGDP.com, Jiang S. et al., 2025).

To effectively prevent and control PEDV, vaccine immunization stands as the foremost approach (Lei et al., 2024; Song and Park, 2012). However, due to the prevalence of multiple PEDV subtypes, vaccines prepared from the classical PEDV GI subtype, such as CV777, can no longer provide effective antigenic protection against the PEDV GII subtype, so the development of typing diagnosis to identify PEDV GI and GII subtypes is of great significance in guiding vaccination (Hou and Wang, 2019; Li et al., 2012; Wang D. et al., 2016). Currently, enzyme-linked immunosorbent assay (ELISA) and quantitative real-time PCR (qPCR) are the main methods for PEDV diagnosis in clinics (Okda et al., 2015; Wang L. et al., 2014). ELISA leverages highly specific antibodies, but cross-reactivity between antibodies targeting the PEDV GI and GII subtypes hinders precise typing. In contrast, qPCR, relying on the base-complementary-pairing and highly specific probes, excels at detecting specific nucleic acid sequences with remarkable sensitivity and specificity, making it an outstanding tool for PEDV typing (Diel et al., 2016). To address the PEDV variation, it is necessary to continually isolate, identify, and sequence prevalent PEDV strains to understand their variation trends. This facilitates the design of broader-spectrum primer and probe sequences, enhancing the adaptability and precision of qPCR. In this study, we isolated a PEDV GIIa subtype (SX0818-2022) from Shanxi Province, China, and sequenced its genome. By comparing the gene sequence of this isolated PEDV strain with known PEDV sequences in the NCBI database, we identified conserved regions that enabled the development of a duplex qPCR assay, which offers accurate detection and identification of both PEDV GI and GII subtypes. The workflow of the study is shown in Figure 1.

## 2 Methods and materials

### 2.1 Virus isolation

The samples comprised intestinal contents and feces sourced from pigs exhibiting clinical symptoms of PED in a pig farm located in Shanxi Province. The isolation was performed in accordance with the previously reported protocol for PEDV isolation, with some modifications (Jiang N. et al., 2018). Upon collection, these samples underwent repeated grinding with liquid nitrogen, followed by mixing with DMEM maintenance solution. Subsequently, they were centrifuged at 12,000 rpm for 20 min and filtered through a 0.22 μm filter. One milliliter of the filtrate was inoculated onto a monolayer of Vero cells, which were then incubated at 37°C for 1.5 h, with the inoculum subsequently discarded. To the inoculated cells, 6 mL of culture medium containing 7.5 μg/mL of trypsin was added, and the cells were further incubated at 37°C in a CO_2_ incubator. Cell lesions were monitored every 12 h for four consecutive days per generation. Finally, the samples underwent a three-cycle freeze-thaw process to eliminate cellular debris, enabling their continued utilization in blinded passages. The Vero cells used in this study were maintained and stored within the Laboratory of Prevention and Control of Important Animal and Zoonotic Diseases at Shanxi Agricultural University.

### 2.2 PCR and indirect immunofluorescence (IFA) identifications

Viral RNA was extracted from viral fluids using the EasyPure Viral DNA/RNA Kit (TransGen Biotech Co., Ltd.). cDNA was synthesized as the first strand cDNA using the HiScript II 1st Strand cDNA Synthesis Kit (Nanjing Vazyme Biotech Co., Ltd.). The primer sequences were PEDV (N)-F: ATGGCTTCTGTCAGTTTTC and PEDV (N)-R: TTAATTTCCTGTGTCGAAGATC. The cDNA and specific primers were utilized for PCR identification.

Identification of the viral fluids were performed according the methods described by Yang et al. (2020). The cells were fixed at room temperature for 30 min using 4% paraformaldehyde and then rinsed three times with PBS. Following fixation, the cells were blocked for 1 h in a PBS solution containing 5% skimmed milk powder. Next, the cells were incubated with the anti-PEDV-N antibody for 1 h at 37°C. After incubation, the cells were washed thoroughly three times with PBS and then incubated with the HRP-conjugated secondary antibody for 1 h at 37°C. Subsequently, the cells were incubated with 4′,6-diamidino-2-phenylindole (DAPI, Solarbio) for 10 min after being washed three times with PBS. Finally, the prepared cells were examined under a fluorescence microscope (Nikon) for IFA identification.

### 2.3 Complete CDS sequencing and analysis

Primers were designed based on the whole gene sequence of the PEDV AJ1102 strain and used to amplify the entire genome of the isolated strain. To facilitate amplification, the entire genome was segmented into 15 overlapping fragments. These neighboring segments were overlapped to ensure complete coverage of the complete coding sequence (CDS). PCR products that were positively identified through agarose electrophoresis were sent to BGI Genomics for sequencing. The sequencing outputs were then stitched together using DNASTAR^1^ software to generate the genome sequence.

To evaluate the genetic relevance, the S protein sequences of various PEDV strains sourced from NCBI^2^ were compared using the neighbor-joining method implemented in MEGA-X.^3^ Additionally, the Clustal-X^4^ and GeneDoc^5^ software were utilized to compare the S protein of the isolated strain against representative strains from diverse PEDV subtypes, enabling the identification and analysis of variant sites specific to the isolate.

### 2.4 Primer and probe design

The S protein sequences of PEDV GI and GII subtypes were retrieved from NCBI. DNAMAN^6^ software were used for multiple sequence comparisons to identify conserved regions within these subtypes. Utilizing Prime 5^7^ and Oligo 7^8^ software, the primers and probes were designed within the conserved regions of the S protein sequences. These primers and probes were synthesized by Sangon Biotech (Shanghai) Co., Ltd.

### 2.5 Standard plasmid construction

Standard plasmid construction was performed with reference to the method described by Ren et al. (2024). The target gene was amplified from the cDNA templates of the isolated strain (SX0818-2022) and the CV777 vaccine strain, utilizing TransStart^®^ FastPfu DNA Polymerase (TransGen Biotech Co., Ltd.) in conjunction with the specific primers designed in 2.4. Subsequently, the amplified gene was inserted into the pEASY^®^-Blunt Zero Cloning vector (TransGen Biotech Co., Ltd.). This recombinant plasmid was then transformed into the *E. coli* DH5α competent cells (Shanghai Tolo Port Biotechnology Co., Ltd.). Positive bacterial cultures were sent to General Biosystems (Anhui) Co., Ltd. for sequencing. Following verification, the positive plasmids were extracted using the TIANprep Mini Plasmid Kit (Tiangen Biotech (Beijing) Co., Ltd.). The concentration of the extracted plasmids was determined using an ultra-micro spectrophotometer (Thermo Scientific NanoDrop One). The number of plasmid copies per μL was calculated via Plasmid copy number (copies/μL) = Plasmid concentration (ng/μL) × 10^−9^ × 6.02 × 10^23^/(Plasmid length (bp) × 660).

### 2.6 Duplex qPCR optimization

A 1:1 mixture of plasmid standards representing the PEDV GI and GII subtypes, each at a concentration of 1.0 × 10^5^ copies/μL, served as the template. The reaction system and procedure were optimized by varying primer and probe addition, as well as annealing temperature. Initially, the optimal concentrations of primers and probes for duplex qPCR were determined. The final primer concentrations ranged from 0.1 μM to 0.6 μM, while the final probe concentrations spanned from 0.05 μM to 0.3 μM. These concentrations were settled by comparing post-reaction fluorescence intensities and cycle thresholds (Ct). The reaction system comprised 10 μL of 2 × Taq Pro HS Probe Master Mix (Nanjing Vazyme Biotech Co., Ltd.), 0.2–1.2 μL each of upstream and downstream primers (both at 10 μM), 0.1 μL-0.6 μL of 10 μM probe, 2 μL of template, and DNase/RNase-free water to bring the total volume to 20 μL. After establishing the duplex reaction system, the annealing temperature was further optimized. Twelve temperature gradients within the range of 50°C–62°C were tested, with the fluorescence intensities and Ct values of each reaction evaluated to identify the optimal annealing temperature. The finalized reaction protocol involved an initial preincubation step at 95°C for 10 s, followed by 40 amplification cycles consisting of denaturation at 95°C for 10 s, annealing at the optimized temperature (50°C–62°C) for 10 s, and extension at 72°C for 20 s. After each cycle, FAM and VIC channel fluorescence signals were detected. All the qPCR experiments were conducted using a LightCycler^®^ 96 instrument (Roche).

### 2.7 Standard curve construction

The concentrations of the PEDV GI and GII subtype recombinant plasmids were standardized to 1.0 × 10^10^ copies/μL, subsequently undergoing 10-fold serial dilutions ranging from 1.0 × 10^9^ copies/μL to 1.0 × 10^2^ copies/μL. Standards of identical concentration gradients were mixed and employed as templates. Amplification was performed according to the optimized duplex qPCR conditions. A standard curve was plotted, with logarithmic concentration serving as the *x*-axis and Ct value as the *y*-axis, facilitating the calculation of the correlation coefficient (*R*^2^) and amplification efficiency (*E*-value) (Malagutti et al., 2020; Ren et al., 2024).

### 2.8 Performance testing of the duplex qPCR

To quantify the sensitivity of the duplex qPCR for detecting the PEDV GI and GII subtypes, a template consisting of a 10-fold serial dilution of standard plasmid, with concentrations spanning from 1.0 × 10^10^ to 1.0 × 10^2^ copies/μL, was utilized. To precisely determine the limit of detection (LOD), 8 additional concentration gradients of standard plasmids, ranging from 90 copies/μL to 20 copies/μL, were sequentially subjected to the assay to identify the lowest plasmid concentration, yielding a positive test result. Each concentration level was replicated 26 times, and the lowest concentration that achieved a positive detection rate exceeding 95% was designated as the reliable LOD. In this study, the positivity threshold was dynamically set by the LightCycler^®^ 96 system.

The specificity of the duplex qPCR was verified by testing different porcine viruses. Specifically, RNA from the PEDV GI subtype, PEDV GII subtype, transmissible gastroenteritis virus (TGEV), and porcine reproductive and respiratory syndrome virus (PRRSV) were extracted and reverse-transcribed into cDNA. Additionally, DNA from the porcine pseudorabies virus (PRV) was extracted and used as a template in the duplex qPCR. Concurrently, conventional PCR was executed using the primers employed in the duplex qPCR. All the viral samples were obtained from laboratory deposits.

The repeatability of the duplex qPCR was evaluated by calculating both inter-assay and intra-assay coefficients of variation (CVs). For this purpose, recombinant plasmids of the PEDV GI and GII subtypes were serially diluted 10-fold to a concentration of 1.0 × 10^9^ copies/μL-1.0 × 10^5^ copies/μL. An equal mixture of plasmids at the same concentration, in a 1:1 ratio, served as the template for the assay. Each reaction was replicated three times within a single experiment, and the entire procedure was repeated across three separate experiments with a 7-day interval between each. The CVs for Ct values were calculated at each concentration level to assess the duplex qPCR repeatability.

### 2.9 Clinical sample detection

To assess the feasibility of the duplex qPCR method established in this study for the detection of clinical samples with reference to Ghosh et al. (2018, 2020). We tested 20 negative samples and 31 positive samples (10 for PEDV subtype GI and 21 for PEDV subtype GII), which were confirmed by conventional PCR and sequencing. Concurrently, conventional PCR was executed using the primers employed in the duplex qPCR. At the end of the reaction, the results were analyzed for consistency with previous diagnoses confirmed. All the samples from pigs were obtained from laboratory deposits.

## 3 Results

### 3.1 SX0818-2022 stain was successfully isolated

**Figure 2:**
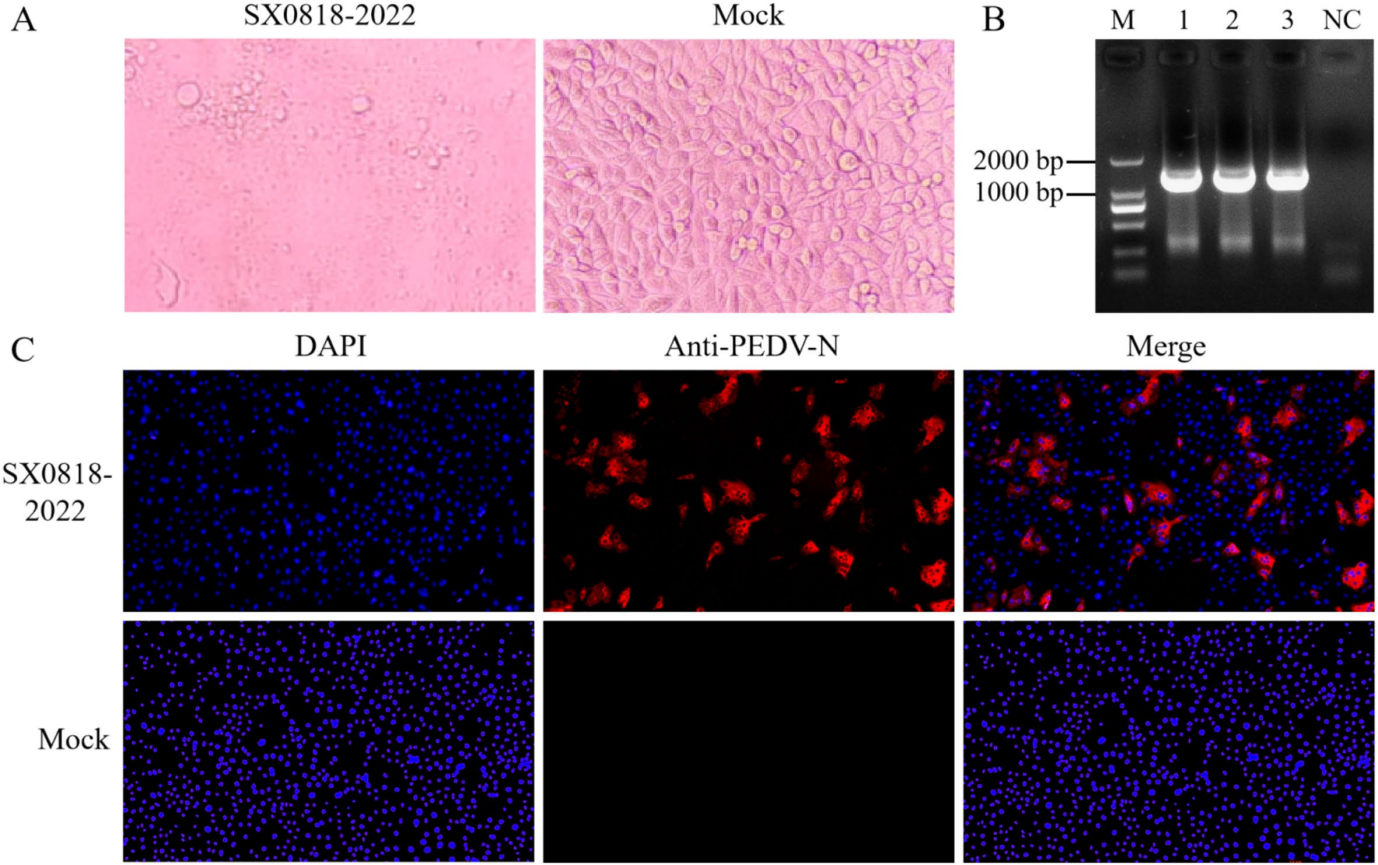
Virus isolation and identification. **(A)** Isolated strain infected cells (SX0818-2022) and control cells (mock) for 24 h. **(B)** PCR results. M: DL2000 DNA marker, 1–3: viral fluids, NC: blank control (water as template). **(C)** IFA results. Fluorescence images of isolated strain infected cells (SX0818-2022) and control cells (mock) with various labels (DAPI: nuclear fluorescence, anti PEDV N: PEDV virus N anti fluorescence, and merge: two channel fluorescence fusion).

In the blind culture, the cell exhibited pronounced PEDV characteristic cytopathic effects (CPE), while the successive blind culture manifested a more advanced CPE, characterized by cells rounding up and nuclei clustering together. Conversely, the control group remained devoid of any CPE, as depicted in Figure 2A.

We used PCR to amplify the PEDV-specific gene, as shown in Figure 2B, and found a specific band at 1,326 bp by agarose gel electrophoresis, indicating the successful isolation of PEDV. The IFA results demonstrated specific immunofluorescence in Vero cells infected with the isolated strain, whereas no fluorescence was observed in the control cells, verifying the isolated strain as PEDV, named SX0818-2022. According to the IFA results, no fluorescence overlap was discernible post-fusion, thereby confirming that the virus was not distributed within the nucleus but mainly in the cytoplasm, as illustrated in Figure 2C.

### 3.2 SX0818-2022 stain belongs to PEDV GIIa subtype according to genome sequencing

**Figure 3:**
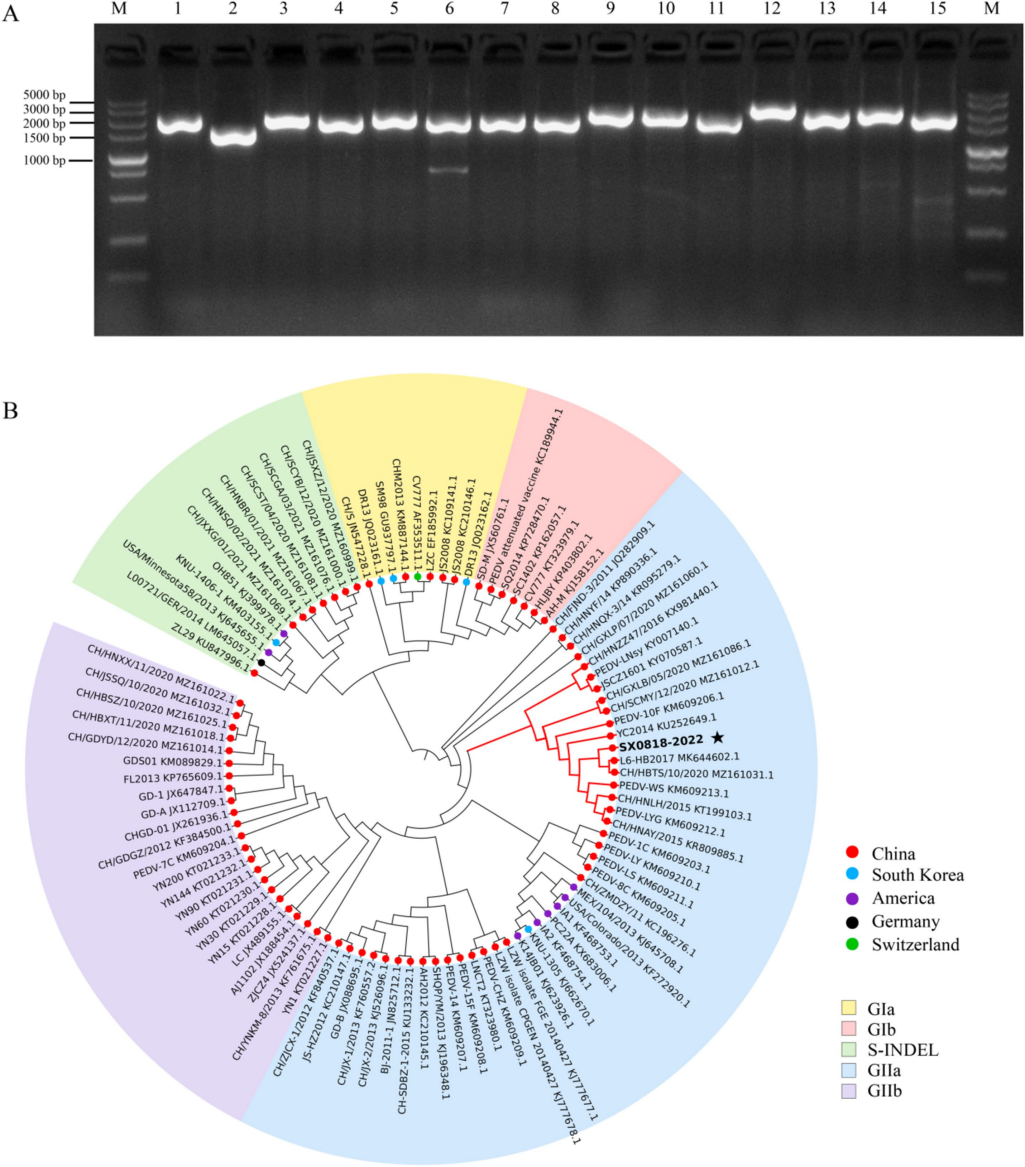
PCR results and phylogenetic tree analysis of genomic sequence. **(A)** Complete CDS amplification map of the isolated strain (M: DL2000 DNA marker, 1–15: genome-wide fragments). **(B)** Phylogenetic tree analysis of the isolated strain based on the S protein.

Upon amplifying the 15 gene sequences of the isolated strain, all bands were consistent with the expected sizes, as shown in Figure 3A. Subsequent splicing revealed that the genome sequence of the isolated strain has a total length of 27,713 bp (complete CDS and partial UTR, GenBank: PQ179480.1). The evolutionary tree presented in Figure 3B illustrates that the isolated strain exhibits a close phylogenetic relationship with strains from Jiangsu (KM609212.1, KU252649, KM609206, and KY070587), Henan (KR809885.1, KT199103.1, and KX981440.1), Heilongjiang (KY007140.1), Shaanxi (MZ161031.1, MZ161086, and MZ161012.1), Zhejiang (KM609213.1), and Hubei (MK644602.1), all of which belong to the PEDV GIIa subtype. Conversely, it demonstrates a relatively distant connection with strains originating from the United States and South Korea, despite these also belonging to the PEDV GIIa subtype.

**Figure 4:**
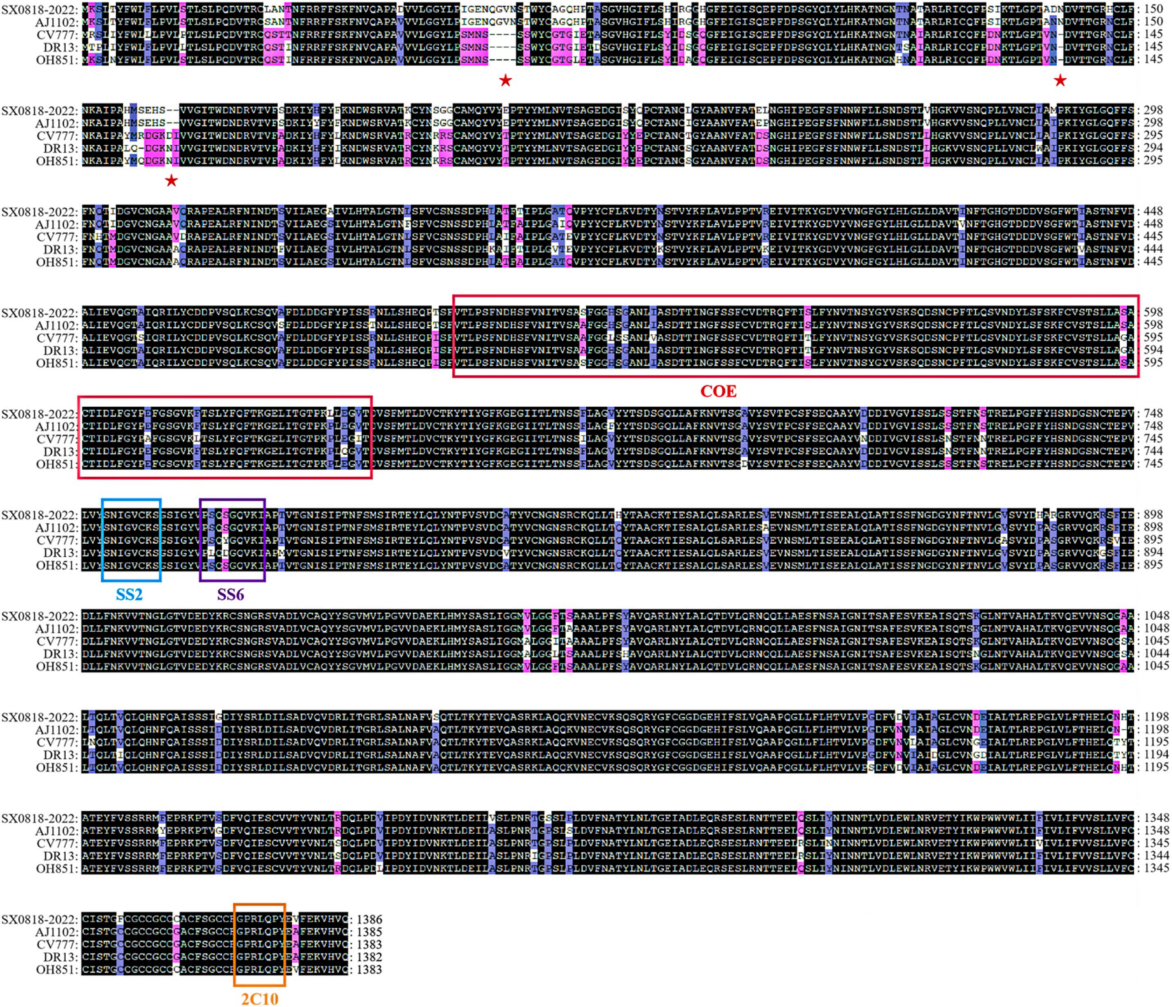
Amino acid sequence comparison of the S protein among the AJ1102, CV777, DR13, OH851, and SX0818-2022 strains. (See Supplementary Table S1 for details).

The S protein amino acid sequences of the isolated strain were analyzed in comparison with other representative strains, such as AJ1102 (AFQ37598.1), CV777 (AAK38656.1), DR13 (AFE85969.1), and OH851 (AHL38184.1). The isolated strain has the identities of 97.47, 92.51, 92.29, and 92.23% corresponding to these representative strains, respectively. Sequence comparison demonstrates that both the isolated and AJ1102 strains have 4 consecutive amino acid (QGVN) insertions at positions aa 59 to aa 62, 1 amino acid (N) insertion at aa 140, and 2 amino acid deletions at aa 163 and 164, compared with the other three strains. Moreover, the isolated strain has 34 amino acid mutations and 1 amino acid deletion compared to the AJ1102 strain. In these strains, there is no amino acid insertion or deletion according to the analysis of the COE, SS2, SS6, and 2C10 antigenic epitopes of PEDV, and it is completely conserved in the SS2 and 2C10 epitope with no mutation. But the isolated strain has 10 amino acid mutations in the COE antigenic epitope and 1 mutation in the SS6 antigenic epitope compared to the CV777 vaccine strain, as shown in Figure 4, indicating the reduced protection of the existing vaccine for the mutated strain.

### 3.3 Primer and probe design

In comparing the S gene sequences of PEDV-GI (GIa and GIb) and PEDV-GII (GIIa and GIIb), we found the identity of the gene sequences were 99.14 and 98.57%, respectively (Supplementary Figure S1). This remarkable sequence conservation indicates that the S gene is highly stable across PEDV subtypes, making it an ideal target gene for developing assays. Using relevant software, primers were designed within the conserved regions of PEDV-GI and PEDV-GII, respectively. We selected primer pairs with higher scores, similar *T*_m_ values and product lengths of about 150 bp. At the same time, probes with *T*_m_ values 5 to 10°C higher than those of the primers and with similar distances from the primers were selected. The selected primers and probes were located in the conserved regions of PEDV-GI and PEDV-GII and were able to specifically recognize the sequences of PEDV-GI and PEDV-GII, respectively. For the PEDV GI and GII subtypes, the TaqMan probes were individually labeled at their 5′ termini with reporter dyes: 5(6)-carboxyfluorescein (FAM) for the PEDV GI subtype and 5-VIC phosphoramidite (VIC) for the PEDV GII subtype. Conversely, the 3′ ends of these probes were labeled with quenchers: 8-bromo-7-hydroxyquinoline 1 (BHQ1) for the PEDV GI subtype and Eclipse^®^ Dark Quencher (Eclipse) for the PEDV GII subtype. The specific primer and probe sequences are detailed in Table 1.

**Table 1:** Primer and probe sequences for the duplex qPCR.

Primer/probe	Sequence (5′ → 3′)	Location	Product/bp
PEDV(S)-GI-F	TCGTTGTTTTGGGTGGTTATC	137–157^a^	162
PEDV(S)-GI-R	CACTAGGATCAAACGGCTC	280–298^a^	
PEDV(S)-GI-P	FAM-AATGCCAATCTCAAAGCCCTGACC-Eclipse	250–273^a^	
PEDV(S)-GII-F	TCAACACTTAGCCTACCACA	43–62^b^	197
PEDV(S)-GII-R	ATACCATGAACGCCACTAGC	220–239^b^	
PEDV(S)-GII-P	VIC-CAGCACAGTACCAAGTTGAATTAACACCC-BHQ1	177–205^b^	

a PEDV strain CV777 spike protein (S) gene, GenBank: JN599150.1.

b PEDV strain SX0818-2022 spike protein (S) gene, GenBank: PQ179480.1.

### 3.4 Standard plasmids for the PEDV GI and GII subtypes were constructed

**Figure 5:**
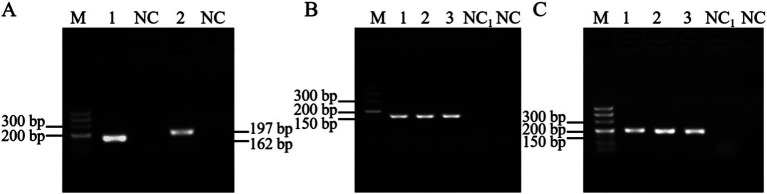
Standard plasmid construction. **(A)** PCR results using cDNA of the PEDV GI and GII subtypes, M: DL500 DNA marker, 1: PEDV GI subtype, 2: PEDV GII subtype, NC: blank control (water as template); colony PCR results of PEDV **(B)** GI and **(C)** GII subtypes, M: DL500 DNA marker, 1–3: bacterial fluids as template, NC: blank control (water as template), NC_1_: blank control (untransformed receptor cells as template).

PCR was performed using the cDNA of the PEDV GI and GII subtypes as templates, resulting in the acquisition of the anticipated fragments of 162 bp and 197 bp, respectively, as depicted in Figure 5A. These fragments were subsequently recovered, ligated into a cloning vector, and transformed into *E. coli*. Following colony PCR screening, positive bacterial fluids corresponding to the PEDV GI and GII subtypes shown in Figures 5B,C were selected for sequencing. The results confirmed the successful construction of the standard plasmids. Finally, the concentrations of the extracted standard plasmids for the PEDV GI and GII subtypes were determined to be 8.28 × 10^10^ copies/μL and 7.65 × 10^10^ copies/μL, respectively.

### 3.5 Duplex qPCR was optimized to pursue high fluorescence intensity and low Ct values

**Figure 6:**
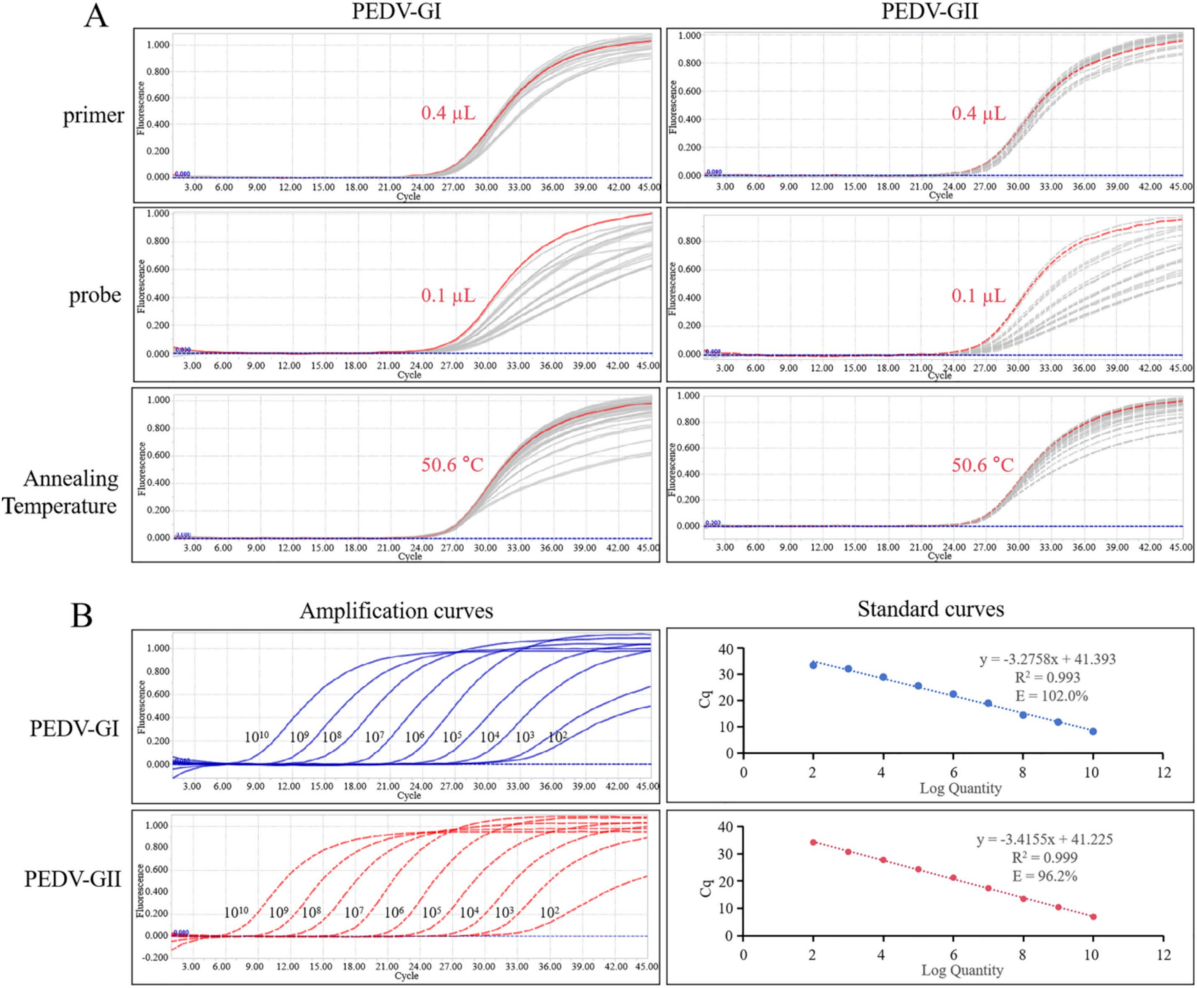
qPCR optimization and calibration. **(A)** Amplification curves of various primers, probes, and annealing temperature for qPCR optimization, the red line represents the optimized condition. **(B)** Amplification curves and standard curves of 10-fold serial dilutions of standard plasmids of PEDV GI and GII subtypes detected by duplex qPCR.

The conditions for the duplex qPCR were optimized using the standard plasmids of PEDV GI and GII subtypes as templates, as shown in Figure 6A. To pursue high fluorescence intensity and low Ct values, the optimal primer volume for both subtypes was determined to be 0.4 μL, achieving a final concentration of 0.2 μM, and the optimal probe volume for both subtypes was determined to be 0.1 μL, yielding a final concentration of 0.05 μM. Further optimization of the annealing temperature revealed that 50.6°C was the optimal temperature for the duplex qPCR.

### 3.6 Standard curves corresponding to PEDV GI and GII subtypes were established

The standard plasmids of PEDV GI and GII subtypes were diluted to a concentration of 1.0 × 10^10^ copies/μL and then mixed in equal proportions. Subsequently, a series of 10-fold dilutions were performed. These mixed standard plasmids, at concentrations spanning from 1.0 × 10^10^ to 1.0 × 10^2^ copies/μL, were utilized as templates for the duplex qPCR. Standard curves corresponding to PEDV GI and GII subtypes could be constructed as depicted in Figure 6B. The standard curves exhibited strong correlation coefficients and high amplification efficiencies, demonstrating the efficacy of the designed primers and probes as well as the suitability of the standard plasmids for use in the assay.

### 3.7 Duplex qPCR was verified with high sensitivity, excellent specificity, and repeatability

Upon utilizing this assay to identify the PEDV GI subtype, a plasmid concentration of 90 copies/μL achieved a perfect 100% positive detection rate (26/26), whereas a lower concentration of 80 copies/μL led to a detection rate of 69.23% (18/26), which did not meet the desired threshold of 95%. Similarly, when targeting PEDV GII subtype, the assay demonstrated a 100% positive detection rate (26/26) at a plasmid concentration of 40 copies/μL, whereas a concentration of 30 copies/μL resulted in a detection rate of 84.62% (22/26), again below the 95% benchmark. Based on these observations, the LOD for the PEDV GI subtype was established at 90 copies/μL and for the PEDV GII subtype at 40 copies/μL, as described in Table 2.

**Table 2:** Sensitivity of the duplex qPCR.

Plasmid	Concentration (copies/μL)	Repeat times	Positive number	Positive rate	95% Positive rate
PEDV-GI	9.0 × 10^1^	26	26	100%	>95%
	8.0 × 10^1^	26	18	69.23%	<95%
PEDV-GII	4.0 × 10^1^	26	26	100%	>95%
	3.0 × 10^1^	26	22	84.62%	<95%

**Figure 7:**
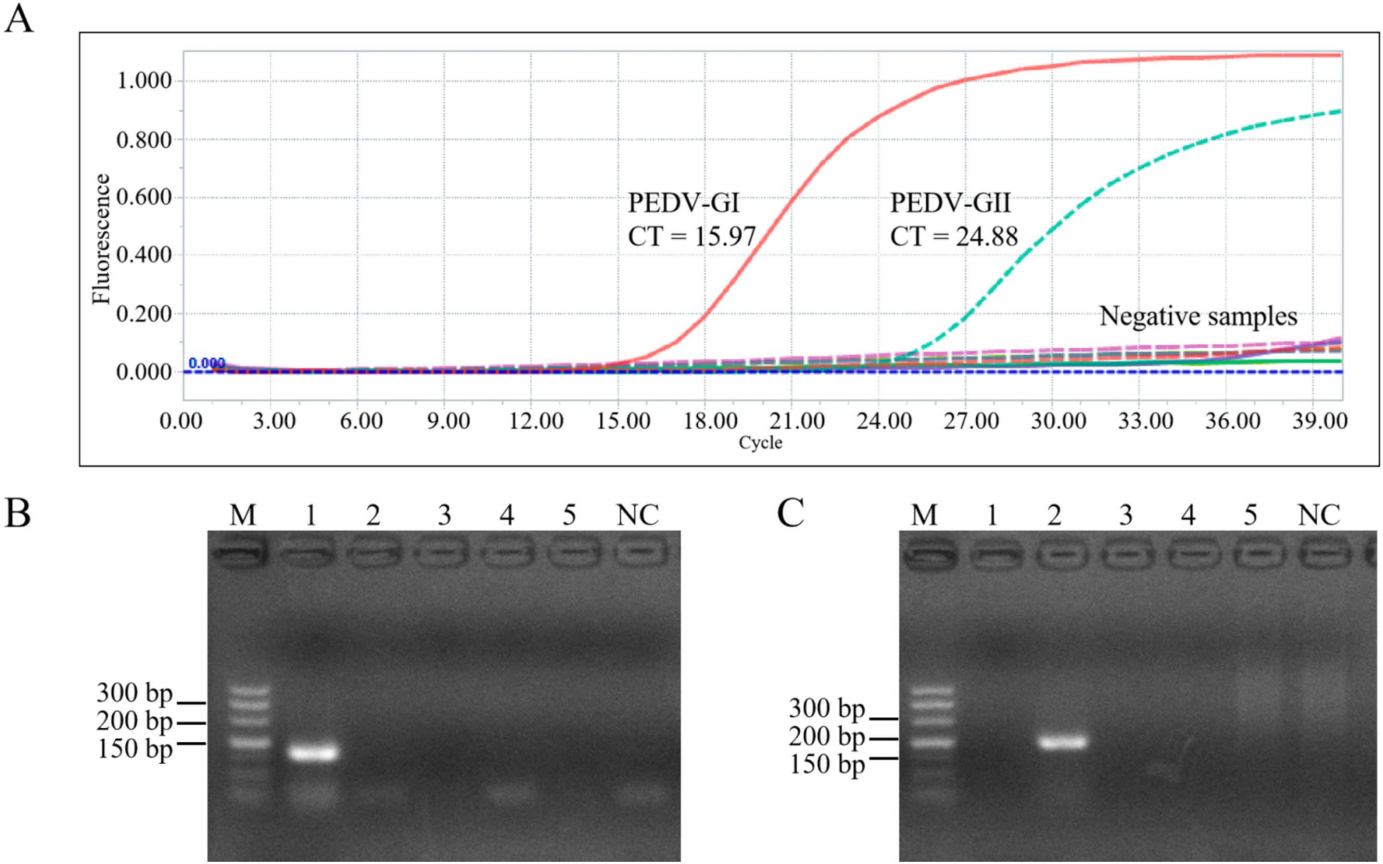
Specificity of the duplex qPCR. **(A)** Amplification curves of plasmid of PEDV GI, plasmid of PEDV GII, TGEV, PRV, PRRSV, and control for specificity verification. PCR results using primers of PEDV GI subtypes **(B)** and PEDV GII subtypes **(C)**, M: DL500 DNA marker, 1–5: PEDV GI, PEDV GII, TGEV, PRV, PRRSV, NC: blank control (water as template).

As depicted in Figure 7, the duplex qPCR exhibited specific detection capabilities for both PEDV GI and GII subtypes, with no amplification curves observed for other porcine viruses such as TGEV, PRV, and PRRSV, nor for the blank control utilizing water as the template. The same results were obtained with conventional PCR.

Table 3 illustrates that when equal concentrations of PEDV GI and GII standard plasmids, spanning a range from 10^9^ to 10^5^ copies/μL, were combined and analyzed using the duplex qPCR, the Ct values demonstrated low intra-assay CVs ranging from 0.07 to 1.18% and inter-assay CVs from 0.23 to 1.89%, both of which remained below 2%. This signifies that the duplex qPCR possessed excellent repeatability.

**Table 3:** Repeatability of the duplex qPCR.

Plasmid	Concentration (copies/μL)	Ct values of intra-assay			Ct values of inter-assay		
		$\bar{X}$	SD	CV (%)	$\bar{X}$	SD	CV (%)
PEDV-GI	1.0 × 10^9^	12.08	0.12	1.00	11.96	0.17	1.39
	1.0 × 10^8^	15.70	0.07	0.47	15.71	0.11	0.73
	1.0 × 10^7^	19.81	0.23	1.18	19.87	0.08	0.39
	1.0 × 10^6^	23.53	0.06	0.26	23.51	0.06	0.23
	1.0 × 10^5^	27.21	0.10	0.38	27.06	0.20	0.72
PEDV-GII	1.0 × 10^9^	10.55	0.02	0.16	10.69	0.13	1.23
	1.0 × 10^8^	14.66	0.03	0.18	14.65	0.11	0.74
	1.0 × 10^7^	19.18	0.12	0.60	18.81	0.36	1.89
	1.0 × 10^6^	22.74	0.02	0.07	22.68	0.17	0.75
	1.0 × 10^5^	26.28	0.13	0.51	26.16	0.37	1.40

### 3.8 Duplex qPCR has been proven to be highly accurate in detecting clinical samples

Using the duplex qPCR method established in this study, we detected 20 previously confirmed negative samples and 31 confirmed positive samples (10 with PEDV subtype GI and 21 with PEDV subtype GII) from pigs. The results of this duplex qPCR, conventional PCR, and the previous confirmatory results were in 100% concordance (Supplementary Table S2), which indicates that the new method is accurate and reliable.

## 4 Discussion and conclusion

In China, porcine epidemic diarrhea virus (PEDV) is the most common pathogen triggering porcine diarrhea and is widely present in most pig farms (Zhang et al., 2024). PEDV belongs to the group of coronaviruses (CoVs), and certain CoVs have continued to adapt and evolve, expanding their host range from wildlife to humans. Since the beginning of the 21st century, humankind has been challenged by a series of outbreaks caused by coronaviruses. Outbreaks such as severe acute respiratory syndrome (SARS), middle east respiratory syndrome (MERS), and severe acute respiratory syndrome coronavirus 2 (SARS-CoV-2), which all have wildlife origins, have posed a significant health threat and economic loss to human society (Shahrajabian et al., 2021). Notably, porcine delta coronavirus (PDCoV), one of the porcine coronaviruses, has also been reported to infect children, triggering acute fever and other symptoms (Lednicky et al., 2021). It is evident that PEDV not only poses a threat to the health of pigs but also lurks as a zoonotic risk. Timely testing is needed to enable timely treatment and control of outbreaks.

The endemic strains of PEDV in China exhibit significant genetic diversity and large differences in virulence and immunity due to immune pressure and high mutation rates in the RNA genome. Prior to 2010, the burden of PEDV in China was sporadic due to the availability of inactivated or attenuated PEDV vaccines. However, since the end of 2010, there has been a large-scale outbreak of PED in China. It was found that mutant PEDV strains will exhibit multiple insertions, deletions, and substitutions of the S protein compared to the vaccine strain GI (CV777). These modifications may alter the antigenicity of the GII strain, rendering the GI vaccine strain ineffective in preventing the large-scale epidemic of PEDV caused by the variant strain in China (Li et al., 2012; Wang D. et al., 2016). In this study, a strain of PEDV virus was isolated and characterized from a pig farm in Shanxi, China, and named SX0818-2022. Compared with vaccine strain CV777, SX0818-2022, had amino acid insertions and deletions of S proteins, as well as many amino acid site mutations, including amino acid mutations in antigenic epitopes, which may alter the antigenicity of the strain. The same GII strains, SX0818-2022 also has 34 amino acid mutations and 1 amino acid deletion compared to AJ1102, as shown in Figure 4. Therefore, current commercial vaccines do not provide complete immunoprotection against PEDV endemic strains (Lei et al., 2024). Notably, some studies have found that vaccine use has influenced PEDV development, increasing the rate of virus evolution (Zhang et al., 2023). In China, the current prevalent strain of PEDV is the GII strain, but studies have shown that the GI strain accounts for 10.1% of the total, and the threat of GI PEDV cannot be ignored (Zhang et al., 2023). We need to develop and use effective assays to monitor the prevalent strains of PEDV. Knowing the locally prevalent strains of PEDV and selecting a vaccine that matches the prevalent strains improves the protection of the vaccine and thus reduces the spread of the virus, which may indirectly affect the rate of evolution of the virus.

In clinical practice, timely detection and accurate diagnosis of the pathogen play an important role in preventing the spread and outbreak of PED. Due to the high similarity of clinical symptoms of different genotypes of PEDV and symptoms and pathological changes caused by viruses such as porcine transmissible gastroenteritis virus (TGEV), porcine delta coronavirus (PDcOV), and porcine acute diarrhea syndrome coronavirus (SADS-CoV), clinical diagnosis cannot be made accurately by clinical symptoms and pathological changes (Yin et al., 2022). Therefore, the establishment of a rapid, accurate, and sensitive diagnostic method for real-time monitoring of PEDV strains is essential for the prevention and control of porcine epidemic diarrhea. There are many methods for PEDV detection, which are mainly categorized into pathogenic culture methods, immunodiagnostic, and molecular biology detection methods. Pathogen culture method requires high laboratory conditions and technology, complicated operation, long detection time, and unable to diagnose in time (Luo et al., 2024). Immunodiagnostic techniques, including indirect ELISA, blocking ELISA, and fluorescent microsphere immunoassays, have limitations in early detection. This is because antibodies are usually not detected in serum until days 6 to 14 after primary infection with PEDV, and antigens may not be detected in fecal samples with very low viral titers (Diel et al., 2016; Okda et al., 2015; Sozzi et al., 2010). For example, PEDV antigen capture ELISA can be detected in the acute phase of the disease and much less frequently in the incubation or recovery phase (Diel et al., 2016; Sozzi et al., 2010). Furthermore, immunodiagnostic techniques struggle to distinguish between different PEDV genotypes due to significant antigenic cross-reactivity between the two PEDV subtypes (Wang X. et al., 2016). In recent years, with the rapid development of molecular biology technology, molecular diagnostic tests have become the method of choice for the diagnosis of PEDV in view of the sensitivity and specificity of the diagnosis and the rapidity of the results (Zhao et al., 2014).

To date, several diagnostic methods have been developed and used to detect PEDV, including gel PCR, loop-mediated isothermal amplification (LAMP), and qPCR. Ishikawa et al. (1997) designed primers based on the M gene of PEDV and developed a conventional RT-PCR assay with a LOD of 100 TCID50/sample. Kubota et al. (1999) designed two pairs of PCR primers based on the N gene of PEDV, which led to the development of a nested PCR assay. Gel PCR is cumbersome and only allows qualitative detection. In view of the high mutation rate of PEDV, the above two developed methods are no longer suitable for the detection of the current epidemic strains. Therefore, it is crucial to emphasize the isolation of the virus and pay close attention to its mutation trends, so as to adjust the gene regions based on which the assay is performed. Ren and Li (2011) designed six primers to amplify the N gene of PEDV, as well as Yu et al. (2015) designed four primers to amplify the M gene of PEDV, and established RT-LAMP methods, respectively, which both showed higher sensitivity than gel-based RT-PCR and ELISA. The complex process of LAMP primer design, which usually involves 4–6 primers, has resulted in extremely efficient amplification and significantly higher yields. However, the method is prone to non-specific amplification due to amplicon cross-contamination (Asadi and Mollasalehi, 2021). Currently, the LAMP technique is mainly used to detect infection with PEDV, and there are still many technical challenges to develop assays that can simultaneously distinguish between its different genotypes. At present, qPCR is the mainstream technology for viral nucleic acid detection. Of course, compared with assays such as immunochromatographic test strips and isothermal amplification, qPCR has the drawbacks of high cost of acquisition of instrumentation and not easy to be carried around, long detection time (usually several hours), and the need for professional testing, which limits its application in clinical diagnostic scenarios that require rapid response. However, due to its advantages of low contamination, fast reaction speed, high sensitivity, and especially for multiplexed detection, qPCR is still widely used in research and diagnostic fields (Arya et al., 2005).

To address the challenges in the diagnosis and control of PEDV, we have developed a new qPCR assay based on a TaqMan probe, capable of identifying common subtypes: GI-a, GI-b, GII-a, and GII-b. In designing primers and probes specific for PEDV GI and GII subtypes, we referred to a large number of known S gene sequences as well as the sequence of a strain of PEDV GIIa subtype isolated and identified in this study. The S gene is relatively conserved in PEDV, but there are sufficient sequence differences between the GI and GII types to enable the design of primers and probes that distinguish between the GI and GII subtypes. In selecting primer sequences, we prioritized regions that are highly conserved in the target sequence and divergent in the non-target sequence. We utilized Prime 5 and Oligo 7 software to select primer pairs with moderate GC content, similar *T*_m_ values and high amplification efficiency. For the design of the probes, we chose probes that were at a similar distance from the primer, would not form a dimer with the primer pair, and had a *T*_m_ value that was more than 5°C higher than that of the primer pair in order to optimize signal generation and detection sensitivity. After the primer probes were synthesized, we performed experimental validation using the isolate strain (SX0818-2022) and the CV777 vaccine strain to ensure that the designed primer probe sequences could accurately differentiate between PEDV GI and GII subtypes.

Currently, qPCR assays for PEDV have been reported. For instance, Zhou et al. (2017) developed a highly sensitive TaqMan real-time RT-PCR method for detecting PEDV, with an analytical sensitivity of 10 copies/μL. Li et al. (2023) developed a multiplex qRT-PCR assay capable of simultaneously detecting PEDV, TGEV, and PDCoV, with a detection limit of 2.95 × 10^0^ copies/μL for each virus. Despite the high sensitivity and specificity of these methods, they are primarily designed to identify the presence of PEDV infection rather than discerning the specific subtype. Previously reported assays have also been able to distinguish between different subtypes of PEDV. For instance, Su et al. (2018) established a qPCR, which achieved the differential diagnosis of classical and variant PEDV with a sensitivity of 4.8 × 10^2^ copies/reaction. Additionally, according to two sets of primers and probes based on the PEDV S protein, Zhao et al. (2014) developed a multiplex qPCR, which enabled the detection of mutant and classical strains with a sensitivity of 5.0 × 10^2^ copies/reaction. We have also successfully developed a duplex qPCR assay that not only effectively detects PEDV, but also differentiates between GI and GII subtypes with sensitivities of 90 copies/μL (180 copies/reaction) and 40 copies/μL (80 copies/reaction), respectively. Clearly, our assay has higher sensitivity compared to the reported techniques described above. Moreover, the duplex qPCR was verified with excellent specificity and repeatability and was validated in 51 local clinical samples. Therefore, the proposed duplex qPCR is especially suitable for domestic prevalent PEDV detection and typing. Based on the advantages of PEDV typing capability, high detection sensitivity, and domestic detection, we envision that this proposed duplex qPCR is a promising tool for PEDV diagnosis, prevention, and control.

The detection method established in this study also has a limitation. The detection capability against S-INDEL subtypes was deficient due to the fact that our primer and probe designs were mainly based on the S gene sequences of GIa, GIb, GIIa, and GIIb of PEDV. The S-INDEL subtypes are genetically different from the rest of subtypes of the GI and GII subtypes due to the presence of a spectrum of base insertions and deletions, which may have led to our primer and probes were not effective in recognizing the S-INDEL subtypes. Therefore, in order to achieve comprehensive detection of all PEDV subtypes, we will further improve our technology and development methods, including the optimization of primer and probe design, to enhance the detection of S-INDEL subtypes.

## Data Availability

The original contributions presented in the study are included in the article/Supplementary material, further inquiries can be directed to the corresponding author.

## Footnotes

^1^ https://www.dnastar.com

^2^ https://www.ncbi.nlm.nih.gov/

^3^ https://www.megasoftware.net

^4^ https://clustalx.software.informer.com

^5^ https://nrbsc.org/gfx/genedoc/index.html

^6^ https://www.lynnon.com/downloads.html

^7^ https://www.premierbiosoft.com

^8^ https://www.oligo.net/demo-downl.html
