# Supplementary material for: Duplex qPCR for detecting and differentiating porcine epidemic diarrhea virus GI and GII subtypes
Source: Front Microbiol. 2025 Jan 22;16:1475273. doi: 10.3389/fmicb.2025.1475273 (PMC11794799; doi:10.3389/fmicb.2025.1475273)
Supplement: Supplementary file 3 [file Image_1.pdf]

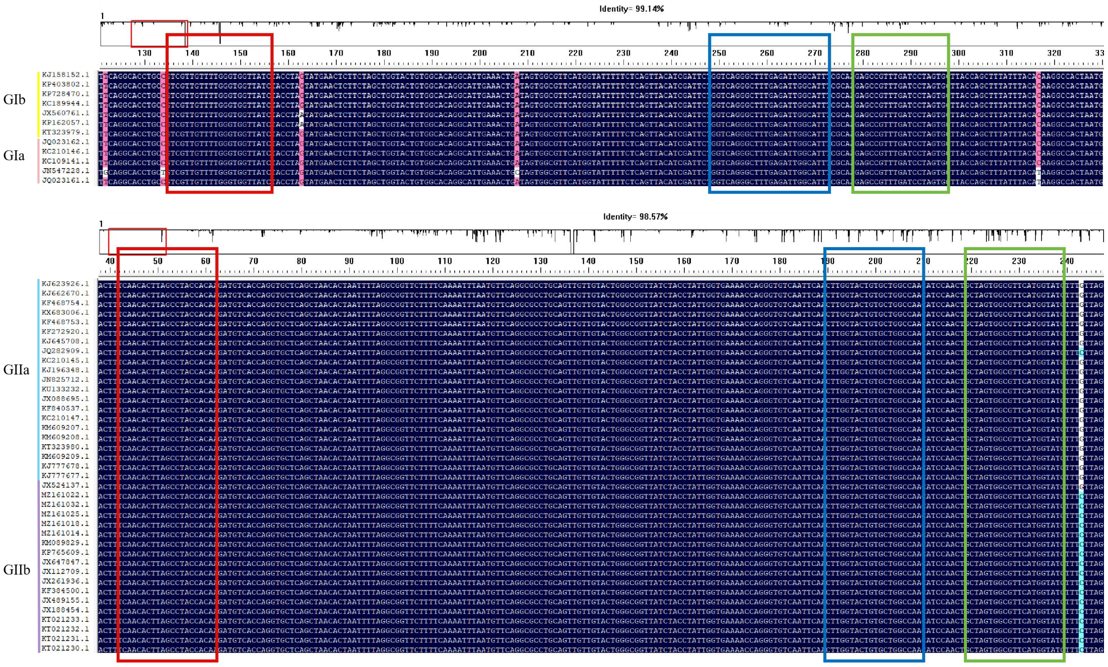

Figure S1: Partial Sequence Alignment of Different Subtypes of PEDV. The red boxes indicate the positions designed for the upstream primers, the blue boxes denote the positions for the probe, and the green boxes mark the positions for the downstream primers.
